# The Origin and Dispersal of the Domesticated Chinese Oak Silkworm, *Antheraea pernyi*, in China: A Reconstruction Based on Ancient Texts

**DOI:** 10.1673/031.010.14140

**Published:** 2010-10-15

**Authors:** Yanqun Liu, Yuping Li, Xisheng Li, Li Qin

**Affiliations:** ^1^ Department of Sericulture, College of Bioscience and Biotechnology, Shenyang Agricultural University, Shenyang 110161, China; ^2^ Sericultural Institute of Liaoning Province, Fengcheng 118100, China

**Keywords:** Chinese oak silkworm, *Antheraea pernyi*, artificial rearing, historic record

## Abstract

Sericulture is one of the great inventions of the ancient Chinese. Besides the mulberry silkworm (*Bombyx mori*), Chinese farmers developed rearing of the Chinese oak silkworm (*Antheraea pernyi*) about 400 years ago. In this paper, the historic records of the origins and dispersal of the domesticated Chinese oak silkworm in China are summarized. The first document with clearly recorded oak silkworm artificial rearing appeared in 1651, although Chinese oak silkworm was documented in about 270 AD. All of the evidence in the available historic records suggests that the domesticated Chinese oak silkworm originated in central and southern areas of Shandong Province in China around the 16th century, and then was introduced directly and indirectly by human commerce into the present habitations in China after the late 17th century. The results strongly support the hypothesis that only one geographically distinct event occurred in domestication of the modern Chinese oak silkworm.

## Introduction

China is an ancient country with a very early agricultural history and the concomitant development of knowledge of entomology took place over a long period of time (Chou 1980; Zou 1981). Sericulture is one of the great inventions of the ancient Chinese. The mulberry silkworm, *Bombyx mori* Linaeus (Lepidoptera: Bombycidae) was successfully domesticated to produce the raw silk used for weaving by Chinese farmers about 5,200 years ago (Chou 1980; Goldsmith et al. 2004). Many people have heard of the mulberry silkworm, however few know much about the Chinese oak silkworm, *Antheraea pernyi* Guérin-Méneville (Lepidoptera: Saturniidae) that also originated in China.

The Chinese oak silkworm is one of the most well-known wild silkworms along with the Japanese oak silkworm, *A. yamamai* Guérin-Méneville, and the Indian oak silkworm, *A. mylitta* Hübner. The Chinese oak silkworm starts its life as a relatively larger egg that is about 0.01g in weight. Unlike the mulberry silkworm egg that can remain dormant, the Chinese oak silkworm egg soon hatches into the silkworm larva. The larva spins a cocoon in which it pupates and emerges from the cocoon as a moth. The moths mate and the female lays several hundred eggs. In southern China, including Henan and Guizhou Provinces, this cycle occurs once a year, but in northern China including the Liaoning, Jilin, Heilongjiang, and Neimenggu Provinces it can occur twice a year. *Antheraea pernyi* enters a pupal diapause in the winter. It feeds on the leaves of *Quercus* and produces a coarse silk. Today it is commercially cultivated mainly in China, India, and Korea for silk production, and is also used as a source of insect food (larva, pupa, moths) for human consumption and for cosmetics in China. About 70,000,000 kg of cocoons (pupae) are produced in China each year, accounting for 90% of world production. The production of cocoons in China's Liaoning Province accounts for 70% of world production. There are currently more than one hundred modern Chinese oak silkworm varieties in China which are divided into four lines based on the larval skin color: yellow, blue, white, and yellow-cyan. This species is also a well-known lepidopteran model system in studies of insect diapause, photoperiod effects, and neuroendocrine regulation (Maida 2000; Chang 2003; Wei 2008) due to its pupal-diapause and large size.

It is well accepted that Chinese oak silkworm was domesticated from its ancestor, a wild type of the Chinese oak silkworm in China (Zhang 1982; Hua 1987). Until now, several wild populations of Chinese oak silkworm have been found in different areas of southern China. A few of the characteristics of the domesticated Chinese oak silkworm have been altered from the wild state. First, the wild type has only a yellow-cyan larval skin color, whereas the domesticated counterpart has the four kinds of skin color mentioned above. Second, the wild type behaves as both univoltine and bivoltine in nature while semidomestic counterparts are exclusively either univoltine or bivoltine. Third, some individuals of wild populations can be kept in pupal-diapause for over two years until the moth emerges out from the cocoon (pupa), whereas no individuals from the domesticated populations can delay the diapause break. These changes presumably were caused by artificial breeding and selection.

About 400 years ago, Chinese farmers had begun to rear Chinese oak silkworm for producing the raw silk as floss. Here successful domestication means that the rearing technology of Chinese oak silkworm, especially including stock selection and reservation, are essentially perfected and stable. Zhang (1982) and Hua (1987) examined the old texts for early records on the origin and radiation of domesticated Chinese oak silkworm in China. Zhang (1991) and Gu (1995) briefly mentioned history of Chinese oak silkworm production. To make it known to wider audience, in this paper we summarize the historic records on the origin and dispersal of domesticated Chinese oak silkworm in China. All these historic records suggest that only one geographically domestication event occurred in producing the modern Chinese oak silkworm. We believe that it is worth doing phylogenetic and geographic analyses to provide new insights into the origin and evolution of the domesticated silkworm species.

### The earliest records of the Chinese oak silkworm

The first document with clearly recorded oak silkworm is *Guang Zhi*, a book on ancient agriculture in China, written by Guo Yigong in about 270 AD. The description is as follows: “There has oak silkworm eating the leaves of oak tree, and the cocoon produced by it can be used as floss.” The name of the oak silkworm comes from this book. However, this book only mentioned the collecting of the cocoons of Chinese oak silkworm from oak trees to make floss, rather than artificial rearing of oak silkworm.

Another document that records wild silkworms is *Gu Jin Zhu*, a book that explains many aspects of ancient and contemporary China; it was written by Cui Bao during the Western Jin Dynasty (265–340 AD). In this book he writes, “At the fourth year of Emperor Yongguang during the Han Dynasty (40 BC), there had wild silkworms emerging in Dongmou Mountain of Donglai County (now Mouping County in Shandong Peninsula). The wild silkworms become cocoons. The cocoons gave birth to moths that produced eggs on stone. About 10,000 Dan (135,000 kg) cocoons were collected to make use of raw silk as floss.” This document is commonly cited and has gained widespread acceptance. In published papers the wild silkworm discussed in this book was considered to be the Chinese oak silkworm (Zhang 1982; Chen 1994; Gu 1995). It seems, therefore, that the collection of Chinese oak silkworm cocoons from the field to make floss dates back to at least 40 BC. However, no mention of artificial rearing of wild silkworms is made in this book.

During the period between the Jin Dynasty and the early Ming Dynasty (about 265–1443 AD), many records about wild silkworms (most likely Chinese oak silkworm) becoming cocoons appeared in different documents in areas including the present AnHui, Hebei, Henan, Hunan, Jiangxi, Liaoning, Shandong, and Shanxi Provinces. At that time, seeing a wild silkworm becoming a cocoon was often considered a good omen because it was not often seen. It should be noted that the records about wild silkworms do not all pertain to Chinese oak silkworm. At least five wild silkworm species including the oak silkworm, *A. pernyi*, samia silkworm, *Samia cynthia*, salix silkworm, *Actias selene*, maple silkworm, *Eriogyna pyretoum*, and tricuspid silkworm *Bombyx mori* (but feeding on *Cudrania tricuspidata* leaves) are described by Wang Yuanting in his book *Ye Can Lu*, the Notes of Wild Silkworms, published in 1902. The book *Jin Shu*, the history of Western and Eastern Jin Dynasties, published in 648 AD noted, “At the seventh year of Emperor Taikang (287 AD), wild silkworms were found being distributed around the Dongmou Mountain (now Mouping County in Shandong Peninsula)” Shen Yue (441–513 AD), a wellknown historian during the Nan Dynasty, noted in the book *Song Shu*, the History of Song Dynasty (420–479 AD): “At the sixteenth year of Emperor Yuanjia (439 AD), there were wild silkworm cocoons in Yuanling County (now Xuancheng County in Anhui Province). The cocoons like hen egg in size, were distributed around the valley, and become more and more in the following years.” The book *Jin Shi*, the History of the Jin Dynasty (1115–1234 AD), written in 1343 said “Wild silkworm cocoons appeared in Jinzhou area of Liaoning Province in July 1125.” In the book *He Nan Tong Shi*, the History of Henan Province, written in 1678 – 1735, it is mentioned that wild silkworms become cocoons at Runing Mountain in May 1403. The book *Nan Chang Fu Zhi*, the Records of Nanchang District in Jiangxi Province, written in 1873 by Xu Yingheng and Wang Zhifan noted, “In 1443 there were wild silkworm becoming cocoon in Jiangxi Province.” This is the latest document that records this phenomenon as a good omen. Notably, *Nong Sang Ji Yao*, a guidance book for agricultural production (especially mulberry silkworm rearing in North China), and *Wang Zhen Nong Shu*, a book on the agricultural technology for North and South China written by Wang Zhen, are two largescale comprehensive agricultural books written in the Yuan Dynasty (1271–1368 AD) and published in 1273 and 1313, respectively. However, they contained no records about the oak silkworm. No record of wild silkworm artificial rearing appeared during the period, prompting us to conclude that the Chinese oak silkworm had not been artificially reared prior to 1443.

### Origin of the domesticated Chinese oak silkworm

The first document to clearly record oak silkworm artificial rearing technology is *Shan Can Shuo*, Talking about Wild Silkworms, written in 1651 by Sun Yanquan (1613–1674) who was the prime minister during early Qing Dynasty. It is a section in *Nan Zheng Ji Lue*, the Records on Crusage for the South Ming Dynasty. In the book, the author describes in detail the production of Chinese oak silkworm and technology for artificial rearing which he saw at the Shimen Village of Zhucheng County in Shandong Province. Some of his descriptions are as follows,: “There are lots of oak trees in oak silkworm field that are located on the hillside. The ant silkworms (small silkworms) are placed sporadically on the oak trees. Silkworms should be transferred to other oak trees when they eat up the leaves. The silkworms are big, strong, and suitable to grow in the field, however sometimes they will be harmed by drought and by birds. The cocoon it produces is about 6 cm in length with a brown color unlike mulberry silkworm cocoon that is yellow or white. The cocoons like hen egg in size are hanging on the tree.” This book, *Shan Can Shuo*, is the earliest known monograph on artificial rearing of Chinese oak silkworm to date (Yu 1987).

Another document that clearly recorded oak silkworm rearing is *Meng Yin*, a poem written in 1643–1661 by Zhang Gangsun. He noted, “It is very difficult to raise oak silkworms because it will take a long time to harvest. Silkworm eats the leaves of oak tree and drinks the early morning dew. To care silkworm, the people go to field very early and go home very late. Both ant and sparrow will harm the silkworm. People have to use slingshot to throw the soil-pill for driving away the sparrow every day, even in stormy weather. People do not dare have some relaxation because they depend on it for living.” This paragraph offers us a clear picture of the hard-work involved in the production of Chinese oak silkworm in Mengyin County of Shandong Province.

The two historic records indicate that artificial rearing technology of Chinese oak silkworm, including stock selection, stock reservation, and silk reeling, had reached a considerably high level at that time in central and southern areas of Shandong Province, including Zhucheng and Mengyin Counties. These records show that the oak silkworm industry had been developed into an important status among local industries. From then on, oak silkworm rearing becomes a true industry that means silkworm people achieve the huge change from collecting the wild cocoons to producing the cocoons.

It is difficult to verify when Chinese oak silkworm was successfully domesticated in Zhucheng of Shandong Province. After 1443, wild silkworms becoming cocoons had not been recorded as a good omen, probably showing that the oak silkworm management was realized sometime later. Zhang (1982) guessed that the rough date of the domesticated oak silkworm was after the midterm Ming Dynasty (around the 16th century).

During the period 1443 — and 1651 AD, there are three documents with records of the oak silkworm that should be considered. In the book *Nan Yang Fu Zhi*, the Records of Nanyang District in Henan Province, compiled from 1522–1566, it is mentioned that “There are two kinds of silks: mulberry silkworm silk and oak silkworm silk. The oak silkworm silk is produced by Nanshao, Zhenping, and etc., among them Nanshao produces the most oak silkworm silk.” Today, Nanshao County of Henan Province still produces the most Chinese oak silkworm silk in South China. The book *Ning Qiang Zhou Zhi*, the Records of Ningqiang District in Shanxi Province, compiled from 1573–1620, noted that oak silkworm cocoons could be used to weave rope. In the book *Li Cheng Xian Zhi*, the Records of Licheng County in Shandong Province, compiled from 1628– 1644, it is described that among all kinds of industries present in the Licheng area the greatest and most lasting benefits are derived from the oak silkworm, so people consider it as one of the main industries. All these do not clearly mention the artificial rearing technology of the oak silkworm; however, we know that all the three areas have considerably high production of cocoons and silk at that time. Furthermore, the publishing years of the three documents are earlier than *Shan Can Shuo* (1651). Hence, we believe that the oak silkworm began being extensively managed at that time in the three areas mentioned above. Why are Henan and Shanxi not the first birthplace of domesticated oak silkworm like central and southern areas of Shandong Province? We suggest that the most likely probability is that the technological problems of oak silkworm rearing and stock production were first overcome by the people in central and southern areas of Shandong Province.

## Dispersal of domesticated Chinese oak silkworm

### From central and southern areas of Shandong Province to Shandong Peninsula

The book *Qi Xia Xian Zhi*, the Records of Qixia County, re-edited in 1879, noted, “Since 1691, people from Zhucheng area taught us to plant oak tree and rear oak silkworm. The output of oak silkworm cocoon here was more compared to other areas in the vicinity, but less than one-tenth of the production in Zhucheng and Yishui areas, that belongs geographically to central and southern areas of Shandong Province.” Wang Yuanting noted in his book, *Ye Can Lu*, “There had not oak silkworm in my hometown, Mouping of Shandong Province. In 1706, Wang Ruyan who was Shandong xuezheng (now education director) began enlisting people from Qingzhou area to teach oak silkworm rearing and supervising to plant oak tree.” Both Qixia County and Mouping County mentioned above belong geographically to Shandong Peninsula. Based on the two historic records, we can conclude that domesticated Chinese oak silkworm stock and rearing methods in Shandong Peninsula were introduced directly from the central and southern areas of Shandong Province.

### From Shandong to Shanxi

The book *Zhu Cheng Xian Zhi*, the Records of Zhucheng County in Shandong Province, written between 1875–1908, noted, “In 1698, Liu Qi, the head of Ningqiang City in Shanxi Province, who was from Zhucheng County of Shandong Province, divided stock cocoons of oak silkworm purchased from Shandong Province to the farmers. Meanwhile, he invited sericulture farmers and silk workers from Shandong Province to teach the rearing method and silk-producing technology.” A similar description also appeared in the book *Ning Qiang Xian Zhi.* Liu Qi first introduced the technology of oak silkworm rearing from Zhucheng of Shandong Province to other Provinces.

### From Shandong to Guizhou

In the book *Zun Yi Fu Zhi*, the Records of Zunyi District in Guizhou Province, is described, “In 1739, Chen Yuxi, the Mayer of Zunyi City, sent his subordinates to buy stock cocoons of oak silkworm and enlisted sericulture master from Shandong Province for raising oak silkworm in Zunyi City. However, he failed because the moths emerged on the way. But in 1741 he did succeed.” We can also obtain the evidence for this case from the book *Gao Zong Shi Lu*, the Records of Emperor Qianlong (1736–1795). In this book there is a letter to the Court written in 1742 by Chen Derong who is Buzhengshi of Guizhou Province (similar to the director of the Civil Affairs Bureau). In this letter he reported “The people use oak tree to rear the spring oak silkworm.”

### From Shandong to Sichuan

The book, *Gao Zong Shi Lu*, noted in a letter to the Court written in 1743 by Jiang Shunlong, who is Anchashi of Sichuan Province (similar to the director of Administration of Justice). Jiang Shunlong reported, “The head of Dayi County, Wang Jun, divides lots of stock cocoons of oak silkworm purchased from Shandong Province to the farmers and teach them the rearing method for two years. Now great success has been achieved.” Thereafter, An Hongde, the head of Mianzhu County, in 1743 and Wang Yingxu, the head of Fengdu County, in 1757 also instructed the farmers to plant oak tree and raise oak silkworm. The two cases appeared in the book *Mian Zhu Xian Zhi*, the Records of Mianzhu County, in Sichuan Province and *Qing Zhou Fu Zhi*, the Records of Qingzhou District in Shandong Province, respectively. Both An Hongde and Wang Yingxu are from Shandong Province.

### From Shandong to Henan

In 1730s, Wang Shijun wrote *Quan Can Ge*, a song to persuade people to rear silkworms that was recorded in *He Nan Tong Zhi*, the Records of Henan Province compiled during the Emperor Yongzheng's reign (1722–1735). In this song it is mentioned that many Henan farmers use oak trees to raise oak silkworms that live on the trees and produce a coarse silk unlike the soft mulberry silkworm silk. This is the earliest record in Henan Province on oak silkworm rearing. The book *Gao Zong Shi Lu* noted the letter to the Court written in 1744 by Shuo Se who is the leader of Henan Province. Shuo Se reported, “Recently many people from Shandong Province come here and carry the cocoons of oak silkworm to rear together with local farmers, and they have succeeded in silkworm stock and rearing method.”

### From Shandong to Liaoning

The book, *Gao Zong Shi Lu*, noted in a letter to the Court written in June 1762 by the provincial government, “There have lots of oak trees on the mountains in Jinzhou, Fuzhou, Xiongyue, and Gaiping areas, all of them belonging to Fengtian Province (now Liaoning Province). These oak trees can be used to raise oak silkworm for cocoon and then to weave the pongee. Now many refugees from Shandong Province come here to put up shanty for residing and raise oak silkworm for a living. They raise silkworms twice a year. After harvest, they produce pongee for a living.” The book, *Ta Zi Gou Ji Lue*, the Records of Tazigou District in Liaoning Province, written in about 1757–1776 AD, noted, “There have many oak trees on the mountain in Tazigou area. The leaves of these trees only can be used to raise oak silkworm, but other use. Firstly many refugees from Shandong Province come here and take silkworm stocks to rear. Now many local people follow their model.”

### From Shandong to Anhui

In the book, *Lai An Xian Zhi*, the Records of Lai'an County in Anhui Province, noted, “In 1766, the County head Han Litang who was from Weixian County of Shandong Province, introduced oak silkworm into Lai'an, and compiled a book, *Yang Can Cheng Fa*, the Method of Rearing Oak Silkworm, based on the book, *Yang Shan Can Cheng Fa* (the Method of Rearing Oak Silkworm written in 1743 by Ke'erjishan).” The book, *Guang Dong Xiang Shan Xian Zhi*, the Records of Xiangshan County in Guangdong Province, also noted, “In 1770, Zheng Ji, the Mayer of Shouzhou City in Anhui Province, purchased stock cocoons of oak silkworm to rear in Shouzhou City.” Zheng Ji's hometown is Xiangshan County in Guangdong Province, so this case was documented in the book.

### From Guizhou to Yunnan, Chongqing

The book, *Xiang Jian Tu Shuo*, the Diagram of Oak silkworm and its Cocoon written by Liu Zuxian, noted, “In 1828, Yunnan farmers purchased stock cocoons of oak silkworm from Anping County of Guizhou Province to rear in Kunming area of Yunnan Province.” In the book, *Qi Jiang Xian Zhi*, the Records of Qijiang District, written from 1782–1850, it is noted that domesticated oak silkworm in Qijiang (the present Qijiang in Chongqing) was introduced from Zunyi of Guizhou Province.

### From Henan to Hunan, Hubei, Zhejiang

Liang (2008) documented that the domesticated oak silkworm was introduced from Henan Province into Hunan, Yingshan, and Xiangyang in Hubei and Yanzhou (the present Jiande County) in Zhejiang in 1905, 1907, and 1909, respectively.

### From Liaoning to Jilin, Heilongjiang, Neimenggu

The book, *The Records of Tussah Varieties in China*, noted that the domesticated oak silkworm was successfully introduced from Liaoning Province into Jilin, Heilongjiang, and Neimenggu in 1907, 1953, and 1958, respectively (Sericultural Institute of Liaoning Province, 1994).

### Conclusions and Remarks

**Figure 1.**
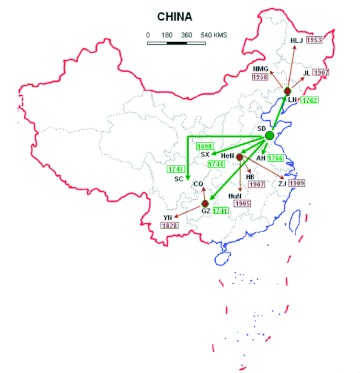
Schematic diagram of origin and dispersal of domesticated Chinese oak silkworm. The green dot refers to the birthplace of domesticated Chinese oak silkworm, and the green arrows refer to the primary dispersal route.The brown dots show the secondary dispersal centers, and the brown arrows show the secondary dispersal route. The boxed numbers accompanying with the dispersal places represent the years of introduction of the domesticated Chinese oak silkworm based on the historic records. AH, Anhui; CQ, Chongqing; GZ, Guizhou; HB, Hubei; HeN, Henan; HLJ, Heilongjiang; HuN, Hunan; JL, Jilin; LN, Liaoning; NMG, Neimenggu; SD, Shandong; SC, Sichuan; SX, Shanxi; YN, Yunnan; ZJ, Zhejiang. High quality figures are available online.

According to these historic records mentioned above, it is very clear that *A. pernyi* was first successfully domesticated in the central and southern areas of Shandong Province in China around the 16th century, and then introduced directly and indirectly by human commerce from central and southern areas of Shandong Province in China into the present habitations distributed over more than half of China after the late 17th century. Figure 1 shows the schematic diagram of origin and dispersal of the domesticated Chinese oak silkworm. The results support the hypothesis that only one geographically domestication event have occurred in the development of the modern Chinese oak silkworm. This is a testable hypothesis for which molecular fingerprinting techniques may be useful, as with the monarch butterfly (Zalucki and Clarke 2004). Preliminary data based on RAPD and ISSR markers carried out in our lab, and with collaborators, suggested the presence of high genetic variation (more than 90% of polymorphism bands) in *A. pernyi* (Liu et al. 2002, 2006; Li et al. 2007). This indicates that the phylogenetic and geographic study of *A. pernyi* can be carried out to further test this hypothesis.

A better understanding of origin and evolution of domesticated species will help to improve domesticated organisms and open opportunities for new domestications. Genetic evidence has revealed many details, not available through historic records, about the origin and early history of domesticated species, including the domesticated silkworm (Pan et al. 2008) and many ungulate domesticated meat animals, e.g. sheep, cattle, pig, horse, goat, dog, donkey, water buffalo, and yaks (Guo et al. 2006 and the references). The domestication place and date of these domesticated species remain unclear; however, the domestication place and date of Chinese oak silkworm are clear. Therefore, we believe that phylogenetic and geographic studies of *A. pernyi* are worth doing to provide new insights into origin and evolution this domesticated species, and we welcome the collaborators to join us in this phylogenetic and geographic study of *A. pernyi.*

The Chinese literature from before 1900 mentioned in this paper is not listed in the references. English titles of papers and books that are only available in Chinese are approximate translations from the original Chinese title by the authors of the paper.
